# *In vivo* evaluation of inter-operator reproducibility of digital dental and conventional impression techniques

**DOI:** 10.1371/journal.pone.0179188

**Published:** 2017-06-21

**Authors:** Emi Kamimura, Shinpei Tanaka, Masayuki Takaba, Keita Tachi, Kazuyoshi Baba

**Affiliations:** Department of Prosthodontics, School of Dentistry, Showa University, Tokyo, Japan; Nanjing Normal University, CHINA

## Abstract

**Purpose:**

The aim of this study was to evaluate and compare the inter-operator reproducibility of three-dimensional (3D) images of teeth captured by a digital impression technique to a conventional impression technique *in vivo*.

**Materials and methods:**

Twelve participants with complete natural dentition were included in this study. A digital impression of the mandibular molars of these participants was made by two operators with different levels of clinical experience, 3 or 16 years, using an intra-oral scanner (Lava COS, 3M ESPE). A silicone impression also was made by the same operators using the double mix impression technique (Imprint3, 3M ESPE). Stereolithography (STL) data were directly exported from the Lava COS system, while STL data of a plaster model made from silicone impression were captured by a three-dimensional (3D) laboratory scanner (D810, 3shape). The STL datasets recorded by two different operators were compared using 3D evaluation software and superimposed using the best-fit-algorithm method (least-squares method, PolyWorks, InnovMetric Software) for each impression technique. Inter-operator reproducibility as evaluated by average discrepancies of corresponding 3D data was compared between the two techniques (Wilcoxon signed-rank test).

**Results:**

The visual inspection of superimposed datasets revealed that discrepancies between repeated digital impression were smaller than observed with silicone impression. Confirmation was forthcoming from statistical analysis revealing significantly smaller average inter-operator reproducibility using a digital impression technique (0.014± 0.02 mm) than when using a conventional impression technique (0.023 ± 0.01 mm).

**Conclusion:**

The results of this *in vivo* study suggest that inter-operator reproducibility with a digital impression technique may be better than that of a conventional impression technique and is independent of the clinical experience of the operator.

## Introduction

A significant change taking place this century is the introduction of digital technology into dental practice; “Digital Dentistry” is becoming more prevalent each year. Recently, digital impression techniques with three-dimensional (3D) intra-oral scanners have been attracting attention gaining in popularity around the world [1]. These intra-oral scanners capture digital images of the dental arches and record occlusal relationships which can directly be used for computer aided design (CAD) and manufacture (CAM) of a dental prosthesis [2–5]. Intra-oral scanners have the potential to replace conventional impression materials for several reasons [6–10]. For example, in contrast to conventional impression techniques, their application clearly simplifies workflow and makes the impression procedure easier and visible for dentists, dental technicians and patients [4,5,11,12]. Furthermore, this method avoids inaccuracies linked to the conventional impression technique, since silicone impression materials are prone to dimensional changes because of on-going chemical reactions and dental stone expands because of secondary reactions. These dimensional changes may result in misfit of a dental prosthesis. In contrast, direct digital scanning of teeth is theoretically not associated with such changes. In fact, several laboratory-based studies report excellent dimensional accuracy and precision of digital impressions when compared with conventional impressions *in vitro* [11,13–17,18]. However, accuracy and precision of the impression may be influenced by various clinical factors, such as the difference in the operator's skill or the patients’ condition, which can only be evaluated by *in vivo* studies. To date, no *in vivo* study investigating the accuracy of this impression technique has been reported. Accuracy can only be evaluated in comparison, preferably with a gold standard; which is not easy to establish in the oral cavity. Regarding precision, there is only a limited number of *in vivo* studies in the literature [19,20] and no study has systematically investigated inter-operator reproducibility of a digital impression technique.

Therefore, this study focused on the inter-operator reproducibility of 3D morphological data captured by a digital impression technique and compared the data to those captured as a result of a convention impression technique *in vivo*. The null hypothesis of this study was that “there is no difference between the inter-operator reproducibilities of the 3D morphological data captured by the digital impression and conventional impression techniques”.

## Materials and methods

### Participants and settings

Twelve participants with a complete natural dentition were included in this study (6 males, 6 females; mean age 26.6±2.0 years). Impressions of the mandibular right second premolar and the first and second molars of these participants were made by two operators with differing levels of clinical experience; one had 3 years of clinical experience (novice dentist) and the other had 16 years clinical experience (experienced dentist). For each participant, each operator employed 2 different techniques, an intra-oral scanner or silicone impression material. All impressions procedures were made in a controlled environment with a room temperature of 24.5±0.9°C and humidity of 30.2±1.7%. In total, forty-eight impressions were made (n = 12). All experimental procedures were approved by the Ethics Committee of Showa University (Approval No. 2013–011). The participants provided written informed consent to participate in this study.

### Digital impression

**Fig 1:**
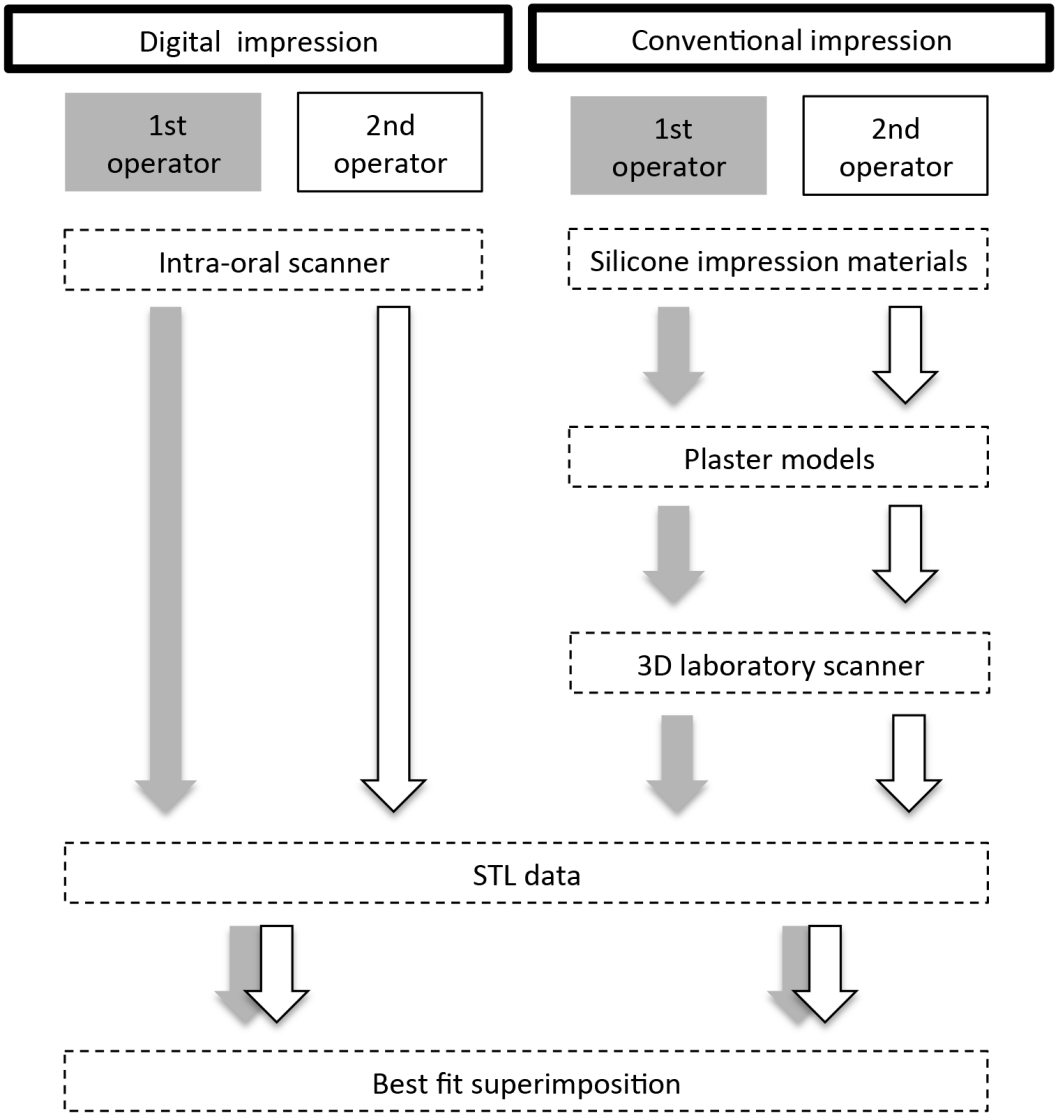
Flow chart demonstrating experimental design of the study.

An intra-oral scanner (Lava COS, 3M ESPE, Germany) was used to make digital impressions. In accordance with manufacturer’s instructions, a dusting powder (Lava COS Powder, 3M ESPE, Germany) was used to pre-treat the surface of the teeth and then digital optical scanning was performed in one continuous scan without any pausing and resuming, starting from the occlusal surface and then moving to the lingual and buccal surfaces. Stereolithography (STL) data obtained by digital scanning were directly exported from the Lava COS system and stored in the laboratory computer (Fig 1).

### Conventional impression

Whole arch conventional impressions were made with a vinylpolysiloxane silicone impression material (addition silicone) (Imprint4, 3M ESPE, Germany) using a double mix impression technique and standard metal stock trays (New IN Toothed all jaw tray, DENSPLY, Japan), which had been carefully chosen to ensure adequate space for impression material. Tray adhesive was not applied to the selected tray, and the heavy body type impression material was injected into the tray using an automatic kneading device. The light type impression material was poured into the corresponding tooth using a syringe, and then the tray was inserted in the oral cavity and pressed to the tooth arch. After a 2-minute setting time, impressions were removed from the oral cavity, disinfected for 10 minutes and stored for 3 hour at room temperature and ambient humidity. Plaster models, fabricated with dental stone (New Fujirock Type IV, GC, Japan) according to manufacturer’s instructions, were then scanned with a dental CAD/CAM 3D laser scanner (D810, 3shape, Copenhagen, Denmark) using the highly accurate protocol for scanning large objects. Scan data of the premolar and molar regions were exported in the STL data format and stored in the laboratory computer (Fig 1).

### Analysis of 3D dataset

First, 3D images constructed from STL data were displayed and trimmed to the tooth shape and gingival margin using appropriate software (PolyWorks, InnovMetric Software, Québec, Canada). These trimmed STL data obtained from novice and experienced dentists were loaded onto a 3D evaluation software platform and superimposed using the best-fit-algorithm method (least-squares method) to match 2 surfaces [17,20]. The STL data from the novice dentist was set as the reference dataset, while those from the experience dentist was the test dataset. Discrepancies between the test and reference data sets (3D image data sets constructed from impressions taken by 2 operators) were analyzed for each impression technique. The software calculates the direction and closest distance of each vertex of the polygon of the test data to the triangle surface of the corresponding polygon of the reference data set. This verification method is routinely used in the industrial field [21–23]. Color mapping of the inter-operator discrepancy for each corresponding measurement point was also displayed for visual inspections [24–27].

The average discrepancies of all measurement points in absolute values were calculated for 2 techniques for each participant. In addition, the average discrepancy for all 12 participants, which represented the inter-operator reproducibility of each technique, was calculated.

### Evaluation of laboratory scanner and intraoral scanner *in vitro*

In order to evaluate the precision of the laboratory scanner, which may influence on the precision of scanned data, laboratory scanning of a cast model was repeated 5 times and the acquired 10 data set pairs were compared using the best-fit-algorithm as described above. These procedures were repeated for the oral scanner in order to test the precision in the laboratory settings.

### Statistical analysis

The average discrepancies of all measurement points in absolute values were calculated and compared between the 2 techniques for each participant by independent t-test. In addition, the average discrepancies for all 12 participants were also compared between the 2 techniques by Wilcoxon signed-rank test. The average *in vitro* reproducibility of the laboratory scanner was compared with that of the oral scanner by independent t-test. All statistical analyses were performed using SPSS version 22 (SPSS Inc., Chicago, IL, USA), and the level of significance was set at 0.05.

## Results

The average discrepancy data for every participant calculated by using the best-fit-algorithm method are summarized in Table 1. Approximately 77,000 polygon points and 9,000 points were obtained from the STL data for the digital and silicone impressions, respectively.

**Table 1:** The number of polygon points analyzed and the average absolute value discrepancy of all measurement points for each participant.

Digital impression technique				
**Participant**	**Mean (mm)**	**Median (mm)**	**95% Cl (mm)**	**Polygon Points**
a	0.0093	0.0062	0.0094, 0.0093	87722
b	0.011	0.0078	0.011, 0.011	78972
c	0.022	0.015	0.022, 0.021	78158
d	0.018	0.013	0.018, 0.017	69792
e	0.015	0.011	0.015, 0.015	81199
f	0.013	0.011	0.013, 0.013	83020
g	0.016	0.012	0.016, 0.016	77710
h	0.015	0.010	0.015, 0.015	72192
i	0.011	0.0069	0.011, 0.011	82013
j	0.013	0.0091	0.013, 0.013	75312
k	0.013	0.0094	0.013, 0.013	76118
l	0.017	0.011	0.017, 0.017	64272
Conventional impression technique				
**Participant**	**Mean (mm)**	**Median (mm)**	**95% Cl (mm)**	**Polygon Points**
a	0.023	0.014	0.023, 0.022	9144
b	0.018	0.013	0.019, 0.018	8998
c	0.032	0.022	0.033, 0.032	9578
d	0.014	0.0089	0.015, 0.014	7488
e	0.025	0.018	0.026, 0.025	9268
f	0.027	0.021	0.028, 0.027	10412
g	0.032	0.020	0.033, 0.031	8564
h	0.023	0.015	0.024, 0.022	8193
i	0.017	0.011	0.018, 0.017	10238
j	0.024	0.019	0.025, 0.024	7543
k	0.023	0.015	0.024, 0.022	8912
l	0.017	0.011	0.017, 0.016	7591

**Fig 2:**
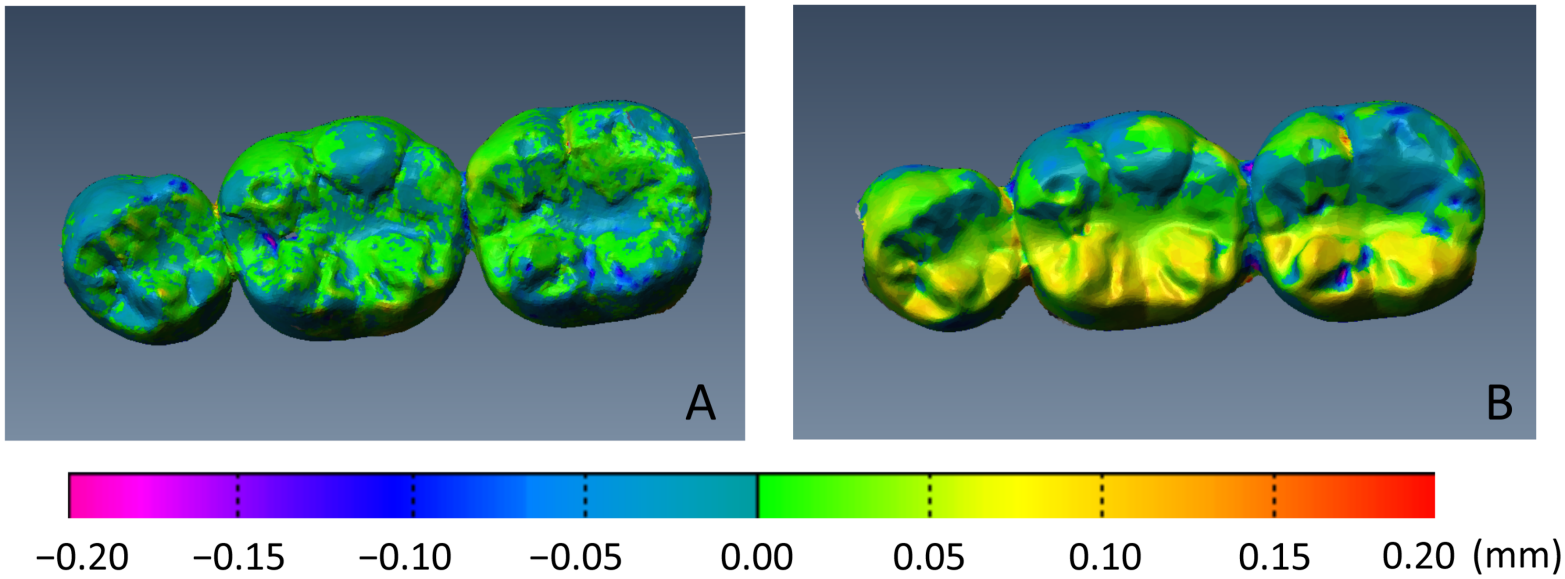
Color mapping for each measurement point (Participant a). A. Digital impression technique. B. Conventional impression technique. Visual inspection of the color mapping data demonstrated a smaller discrepancy (green and blue) between repeated measurements by the two operators for the digital impression technique than for the conventional impression technique (yellow and red).

**Fig 3:**
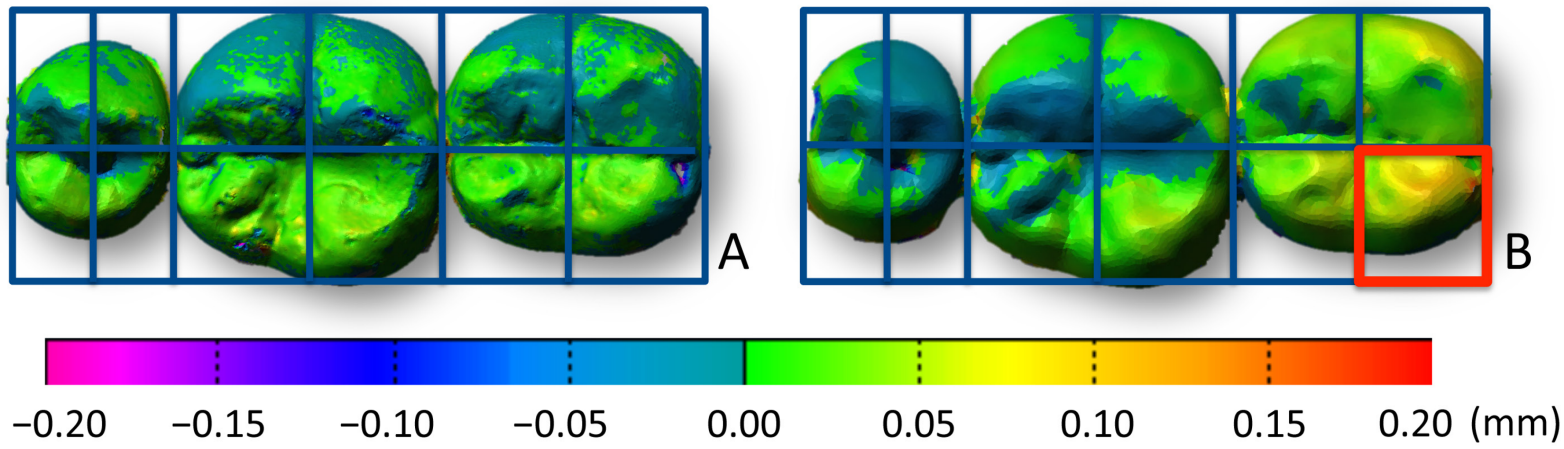
The 3D images were divided into 12 regions and the region with the largest discrepancy was visually identified. A. Digital impression technique. B. Conventional impression technique. The 3D images were divided into 12 regions, and the region with the largest discrepancy was visually identified (red). The largest discrepancy was frequently found on the lingual surface of the second molar for the conventional impression technique (8 out of 12 participants), whereas no such trend was found for the digital impression technique.

Fig 2 shows color mapping of the inter-operator discrepancy between the STL data obtained for each technique (Participant a). Visual inspection of the color mapping data demonstrated a smaller discrepancy between repeated measurements by the 2 operators for the digital impression technique than for the conventional impression technique. In addition, 3D images were divided into 12 regions as shown in Fig 3 and the region with the largest discrepancy visually identified (Participant b). The largest discrepancy was frequently found on the lingual surface of the second molar for the conventional impression technique (8 out of 12 participants), while no such trend was found for the digital impression technique (Fig 3).

**Fig 4:**
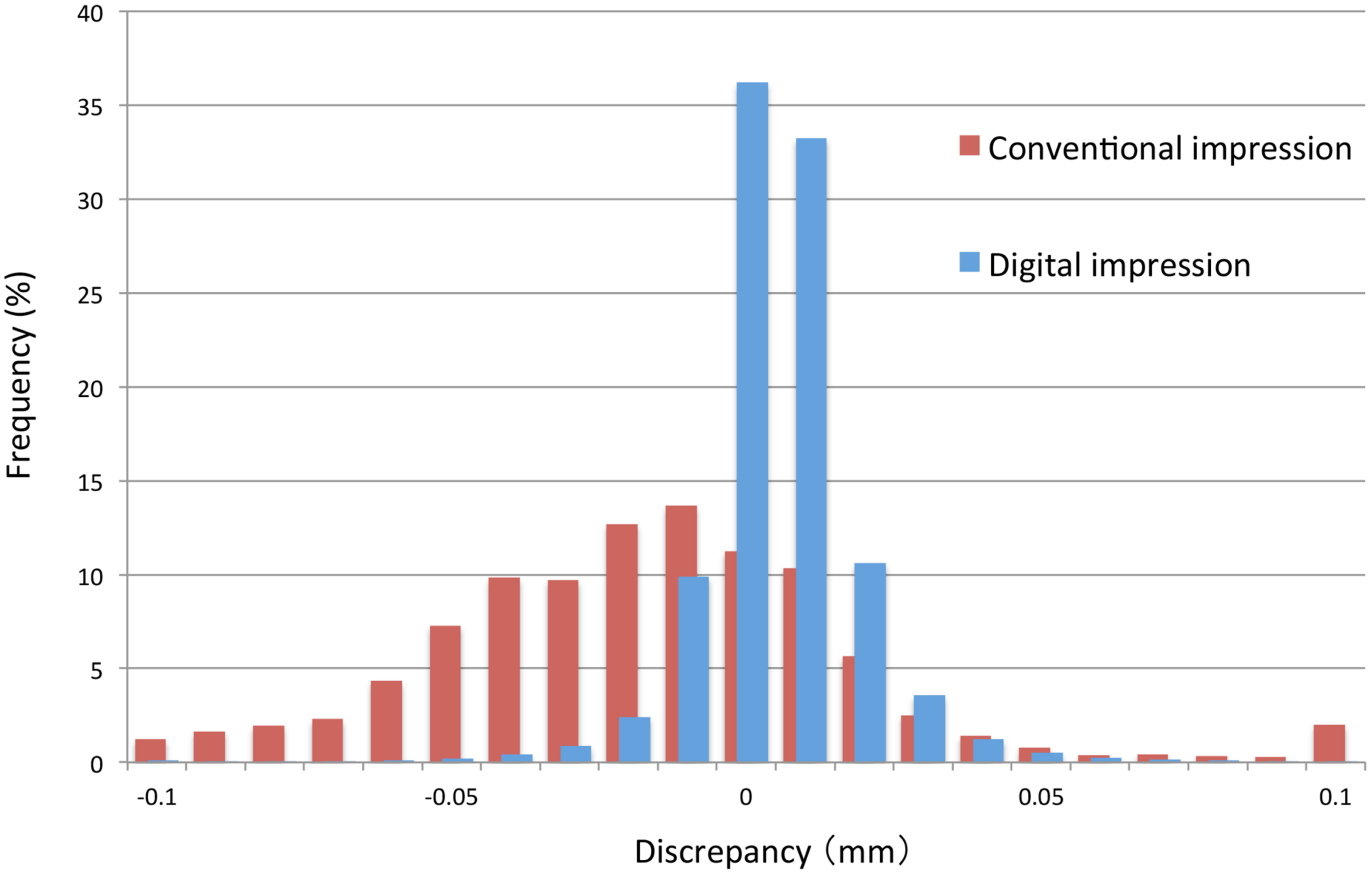
Distribution of dimensional discrepancies between repeated measurements by the 2 different operators for each impression technique (Participant a). Distribution of discrepancy concentrated on zero for the digital impression technique when compared with the conventional impression technique.

**Fig 5:**
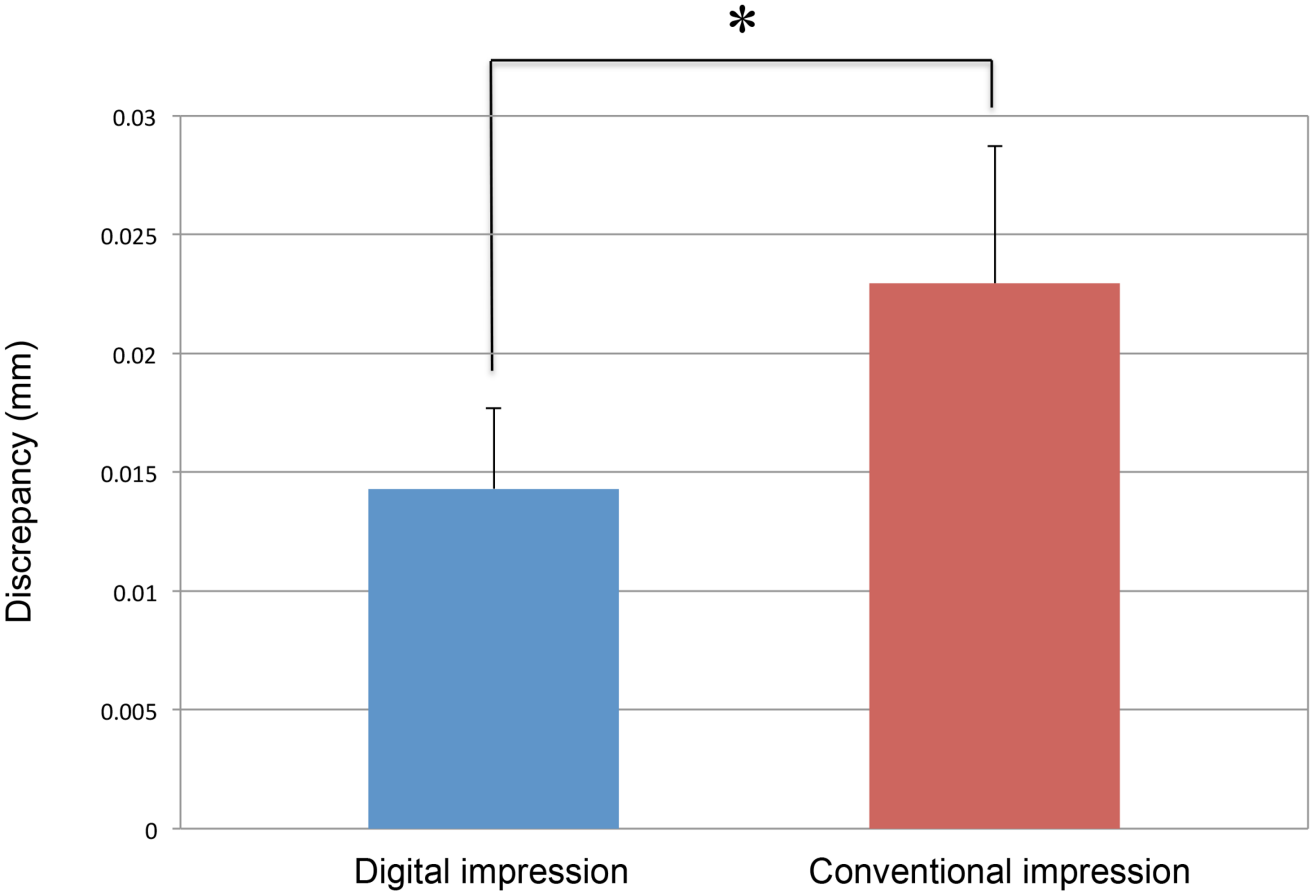
Average inter-operator discrepancy for the 12 participants (Wilcoxon signed-rank test).

Fig 4 shows the distribution of the discrepancies between the two operators for each technique (Participant a). The distribution for the digital impressions was well concentrated on zero compared to the distribution observed with the conventional impression technique. The average discrepancies of all measurement points in the absolute value was significantly lower for the digital impression technique than that for the conventional impression technique in every participant (p<0.05, Table 1). Overall, the average inter-operator discrepancy for the 12 participants with the digital impression technique (0.014 ± 0.02 mm) was also significantly smaller than that of the conventional impression technique (0.023 ± 0.01 mm) (Fig 5).

The average precision of 10 pairs of measurements with the laboratory scanner and the intraoral scanner was 0.007 ± 0.0007 and 0.006 ± 0.0017 mm, respectively, and no significant difference was observed.

## Discussion

### Main finding

These results of this study lead us to reject the null hypothesis of this study that “there is no difference between the inter-operator reproducibilities of the 3D morphological data captured by the tested digital impression and conventional impression techniques”. More specifically, the study results suggest that inter-operator reproducibility of the digital impression technique is higher than that of the conventional impression technique.

### Merit of digital impressions

The merits of digital direct scanning techniques over conventional impression techniques have been well documented as described in the introduction section. Although digital impression is reported to be associated with inevitable inaccuracy sources, such as presence of saliva, movement of the patient’s jaw, or stitching of multiple digital images. One of the most clinically important benefits is that a digital impression is not vulnerable to unavoidable inaccuracies associated with the dimensional changes of impression materials and dental stones that may lead to misfit of dental prostheses.

### Accuracy and precision

Several laboratory-based studies report excellent dimensional accuracy and precision from digital impressions compared to conventional impressions *in vitro* [11, 13–17]. Since the accuracy and precision of an impression are influenced by various clinical factors, *in vivo* clinical evaluations of are vital in order to demonstrate translation of *in vitro* finding to clinical significance *in vivo*. However, accuracy, which represents trueness, is difficult to evaluate *in vivo* because the real dimensions of the test participant, the gold standard (ISO 5725–1) [27,28], cannot be easily measured in the oral cavity of patients. Therefore, *in vivo* evaluations of accuracy are often performed by measuring the fit of final restorations [3,7,20,29,30]. Studies have reported clinically acceptable fit of restorations fabricated using a digital impression technique and when compared to restorations fabricated using conventional impressions [6,31–34]. It should be noted that these accuracy measurements take the entire production process of the restoration into account, but do not exclusively evaluate the impression procedure. Therefore, comparison of the impression techniques themselves is needed.

Precision of impression technique is best assessed by superimposing entire scanned images captured using a given method several times [16,17,20,35]. In this procedure, deviations between the images of the impression at each surface point are determined from computed 3D distances. Such comparison is feasible clinically; however, there is only a limited number of studies that have evaluated the precision of impression technique *in vivo* [19,36] and no study has systematically investigated inter-operator reproducibility. This study should be regarded as the first to accept the challenge of evaluating inter-operator reproducibility of digital impression in a biologically clinically relevant environment.

### Study results

The current study is best compared to the results of Ender *et al*. (2016) who evaluated 2 conventional and 7 digital impression systems in 5 participants [19]. They reported acceptable precision of the digital impression systems *in vivo* and also reported significant variation in the level of precision depending upon the system tested. Our study results are in general agreement. A clear improvement of our study is the higher number of the participants we included and that we conducted a systematic analysis of the inter-operator reproducibility. The latter is particularly clinically important because silicone impressions are notoriously sensitive to the skill and experience level of the clinician who is making the impression [10,12]. Of importance, our study showed significantly better consistency of 3D data made from direct oral digital scanning by two different operators when compared with data from silicone impressions. Since the excellent accuracy and precision of the laboratory scanner has been demonstrated in this study and in others, it is logical to consider that one source of the inconsistency of stone models made from silicone impressions is the dimensional changes of the materials [20,37–41].

Another source of error might be the difference in clinician skill level. Interestingly, the most significant discrepancies between repeated recordings were found on the lingual distal surface of the molar for the silicone impression technique. This finding illustrates well the clinical situation because this region is prone to contamination by saliva and difficult for novice dentists to manage and make an accurate impression. Furthermore, the distal region of second molars might not be fully covered or impression material may not be adequately supported by the impression tray, which propagates distortion of the impression material under the influence of the weight of the plaster during the pouring process. Again, these factors are influenced by the condition of the patient and the skill level of the operator and regarded as impactful reasons why the silicone impression technique exhibits poorer inter-operator reproducibility than digital impression techniques. It should be noted that our study did not examine accuracy but precision and employed only one operator in each clinical experiences, which does not allow drawing a conclusion that the clinical experience was the definitive influencing factor. However, an important finding from our study is that the improved reproducibility of digital impressions compared to silicone impressions, was consistent and independent of participant (Table 1), suggesting that the principal influencing factor may be the inherent dimensional changes associated with the silicone impression technique.

Lastly, although the difference in the average discrepancy between the two techniques was as small as 0.009 mm, which was not considered clinically relevant, our result suggesting better precision with the digital impression method than with the gold standard method might be a good reason for clinicians to apply this method in clinical settings.

### Study limitations

This study exclusively investigated three natural mandibular teeth in healthy participants. We did not investigate prepared abutment teeth, which could be a limitation since both shape and characteristics of a tooth surface may influence accuracy and/or precision of an impression technique. Another limitation might be that this study only tested precision of the technique, whereas clinical relevance might be evaluated by measuring the fit of fabricated prosthesis. We suggest future studies if preliminary studies like ours have demonstrated clinically acceptable inter-operator reproducibility *in vivo*.

## Conclusion

The result of this *in vivo* study suggest that a digital impression technique yields superior reproducibility compared to conventional impression technique. This advantage is independent of an operator’s clinical experience of the operator and of the oral condition of the patient.
